# SAP97 and Cortactin Remodeling in Arrhythmogenic Purkinje Cells

**DOI:** 10.1371/journal.pone.0106830

**Published:** 2014-09-03

**Authors:** Wen Dun, Patrick Wright, Peter Danilo, Peter J. Mohler, Penelope A. Boyden

**Affiliations:** 1 Department of Pharmacology, Center for Molecular Therapeutics, Columbia University, New York, New York, United States of America; 2 The Dorothy M. Davis Heart & Lung Research Institute, The Ohio State University Wexner Medical Center, Columbus, Ohio, United States of America; 3 Department of Internal Medicine, The Ohio State University Wexner Medical Center, Columbus, Ohio, United States of America; 4 Department of Physiology and Cell Biology, The Ohio State University Wexner Medical Center, Columbus, Ohio, United States of America; The Ohio State University, United States of America

## Abstract

Because structural remodeling of several proteins, including ion channels, may underlie the abnormal action potentials of Purkinje cells (PCs) that survive in the 48 hr infarcted zone of the canine heart (IZPCs), we sought to determine the subcellular structure and function of the K_V_1.5 (KCNA5) protein in single IZPCs. Clustering of the Kv1.5 subunit in axons is regulated by a synapse-associated protein, SAP97, and is linked to an actin-binding protein, cortactin, and an intercellular adhesion molecule, N-cadherin. To understand the functional remodeling of the Kv1.5 channel and its regulation in IZPCs, Kv1.5 currents in PCs were measured as the currents blocked by 10 µM RSD1379 using patch-clamp techniques. Immunocytochemistry and confocal imaging were used for both single and aggregated IZPCs vs normal PCs (NZPCs) to determine the relationship of Kv1.5 with SAP-97, cortactin and N-cadherin. In IZPCs, both the sarcolemma (SL) and intercalated disk (ID) Kv1.5 protein are abundant, and the amount of cytosolic Kv1.5 protein is greatly increased. SAP-97 is also increased at IDs and has notable cytosolic localization suggesting that SAP-97 may regulate the functional expression and stabilization of Kv1.5 channels in IZPCs. Cortactin, which is located with N-cadherin at IDs in NZPCs, remains at IDs but begins to dissociate from N-cadherin, often forming ring structures and colocalizing with Kv1.5 within IZPCs. At the same time, cortactin/Kv1.5 colocalization is increased at the ID, suggesting an ongoing active process of membrane trafficking of the channel protein. Finally, the Kv1.5 current, measured as the RSD1379-sensitive current, at +40 mV did not differ between NZPCs (0.81±0.24 pA/pF, n = 14) and IZPCs (0.83±0.21 pA/pF, n = 13, NS). In conclusion, the subcellular structural remodeling of Kv1.5, SAP97 and cortactin maintained and normalized the function of the Kv1.5 channel in Purkinje cells that survived myocardial infarction.

## Introduction

Macromolecular structural and electrophysiological remodeling may underlie the abnormal action potentials (APs) of Purkinje cells that survive in a 48 hr infarcted canine heart (IZPCs). Such changes may lead to the serious ventricular arrhythmias that occur during the first week post MI [1]. AP recordings of the subendocardial Purkinje fibers that survive in the border zone 24 to 48 hr after occlusion have a small degree of rapid phase 1 repolarization when the fibers are driven at slow rates. Pacing at fast rates causes little or no change in the phase 1 repolarization in normal Purkinje fibers, yet it dramatically lengthens the time course of the repolarization of subendocardial Purkinje myocytes that survive in the infarcted heart (see Fig 3.30 of [2]). Whole-cell voltage-clamp experiments have confirmed that the density of I_to_, the transient outward current, in IZPCs is reduced by 51%, while the time course of reactivation of I_to_ is significantly delayed. IZPCs also have a significantly increased density of E4031-sensitive currents compared with that of normal Purkinje myocytes (NZPCs). E4031-sensitive Purkinje IZPC currents differ from those of the normal or infarcted ventricular myocytes (I_Kr_), and their molecular identity is unknown at this time [3]. In same study, 4-AP sensitive basal currents were enhanced in IZPCs.

One K^+^ current that has received increasing attention is I_Kur_ (*KCNA5*); this current has been reported in human atrial myocytes but is absent in human ventricular cells [6]. In the canine ventricle, I_Kur_ currents (defined as currents sensitive to 100 µM 4-AP) have been identified in myocytes [4], [5]; however, Purkinje cells were not studied. Thus, the role of I_Kur_ in both normal and diseased Purkinje cells remains unknown. The goal of this study was to determine the nature of I_Kur_ currents as well as KCNA5 auxiliary subunits, SAP-97, Cadherin and Cortactin, in normal and diseased PCs.

## Methods

This investigation was conducted in strict accordance with the recommendations in the Guide for the Care and Use of Laboratory Animals of the National Institutes of Health (Publication No. 85-23, 1996). The protocol for all animal procedures was approved by the Institutional Animal Care and Use Committee of Columbia University (Permit Number: AC-AAAD1067). Healthy mongrel male dogs (12 to 15 kg, 2 to 3 years old) were used in these studies. Under isoflurane anesthesia (30 mg/kg)and sterile conditions, myocardial infarction was produced by a 2-step total occlusion of the left coronary artery using the Harris procedure [7]. The dogs were treated with lidocaine (2 mg/kg IV) if multiple ventricular beats occurred during the surgical procedure. Two days after the MI surgery, the animals were euthanized using 5–15 mg/kg IV propofol anesthesia; then, the animals were sacrificed, and a cardiectomy was performed. All efforts were made to minimize suffering. Thin strands of subendocardial Purkinje fibers were dissected from the LV subendocardium of the 48 hr infarcted zone and normal non-infarcted hearts and were used to disperse single Purkinje cells (IZPCs and NZPCs) [8] for voltage-clamp experiments and immunocytochemistry.

### Immunostaining and confocal microscopy

NZPCs and IZPCs dispersed from the LV subendocardium were plated on laminin-coated glass chamber slides. Single cells of each to the two types were fixed with 4% paraformaldehyde for 15 minutes, rinsed with PBS (Sigma), permeabilized by 0.7% Triton X-100 (Sigma) for 20 minutes, blocked in 10% normal goat serum for 30 minutes, and then incubated with primary antibodies overnight at 4°C. The cells were rinsed in PBS, incubated with 1∶400 Alexa Fluor 488-conjugated or/and Alexa Fluor 594-conjugated IgG (Molecular Probes) for 1.5 hours at room temperature, and rinsed in PBS. Coverslips were mounted on slides using aqueous mounting medium (Biomeda Corp, Foster City, CA). Signals were viewed using a Red Nikon A1 confocal system (488 nM and 594 nM excitation). Care was taken to view images from the NZPC and IZPC preparations on the same microscope on the same day.

### Antibodies

The antibodies used in this study include rabbit anti-Kv1.5 (1∶100, Alomone labs), mouse anti-SAP97 (1∶100, Santa Cruz), mouse anti-cortactin (1∶200, Millipore), rabbit anti-cortactin (1∶100, Cell Applications), and mouse anti-N-cadherin (1∶100, BD).

### Electrophysiology

Single cells were placed at the bottom of a 0.5-ml tissue chamber,which had been mounted on the stage of a Nikon inverted microscope(Nikon Diaphot, Tokyo, Japan). The myocytes were superfused (2–3 ml/min) with Tyrode's solution containing (in mmol/L) NaCl 137, NaHCO_3_ 24, NaH_2_PO_4_ 1.8, MgCl_2_ 0.5, CaCl_2_ 2.0, KCl 4.0, and dextrose 5.5 (pH7.4). Patch pipettes were made from thin-walled borosilicate glass, and the pipette resistances ranged between 1.0 and 1.5 MΩ when filled with an internal solution containing (in mmol/L) KCl 140, MgCl_2_ 1.0, HEPES 10, Mg-ATP 5,EGTA 10, and Na_2_-phosphocreatine 5 (pH 7.3 with KOH). After the formation of the gigaohm seal, the cell membrane under the pipette tip was ruptured by a brief increase in suction, resulting in the whole-cell recording configuration. A period of 5–10 min was then allowed for intracellular dialysis to begin before the switch to the external recording solution containing (in mmol/L) N-methyl-D-glucamine 144, KCl 5.4, MgCl_2_ 1.0,CaCl_2_ 2.5, HEPES 10, and CdCl_2_ 0.5 (pH 7.3) at 30–31°C. Data were acquired from all studied cells at the same time after the whole-cell recording. Thus, the cells from normal and diseased tissues could be compared. The membrane currents associated with Na^+^/Ca^2+^ exchange were eliminated by the absence of external Na^+^. Outward K^+^ currents were elicited by a 250-ms voltage step to test potentials of −50 to +60 mV from a holding potential of −60 mV at 0.1 Hz after a 10-ms prepulse to −90 mV. *I*_sus_ was measured as the amplitude of the current at the end of the test pulse relative to the zero-current level. The RSD1379 (C9356) (Cardiome Pharma Corp, Vancouver)-sensitive current was defined as the K_v_1.5 current [4].

### Statistics

Values represent the mean ± SE. A value of *P*<0.05 was considered statistically significant. An unpaired *t*-test or paired t-test was used to compare a single mean value between two independent or paired cell groups.

## Results

### Kv1.5 location in NZPCs vs IZPCs

**Figure 1:**
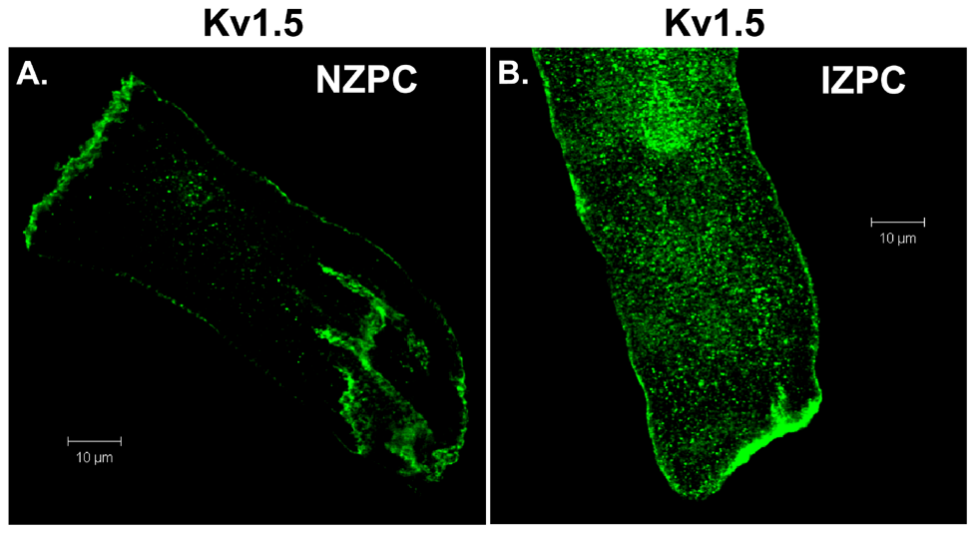
Immunolocalization of Kv1.5 in a single NZPC (A) and IZPC (B). Horizontal scale = 10 µm.

**Figure 2:**
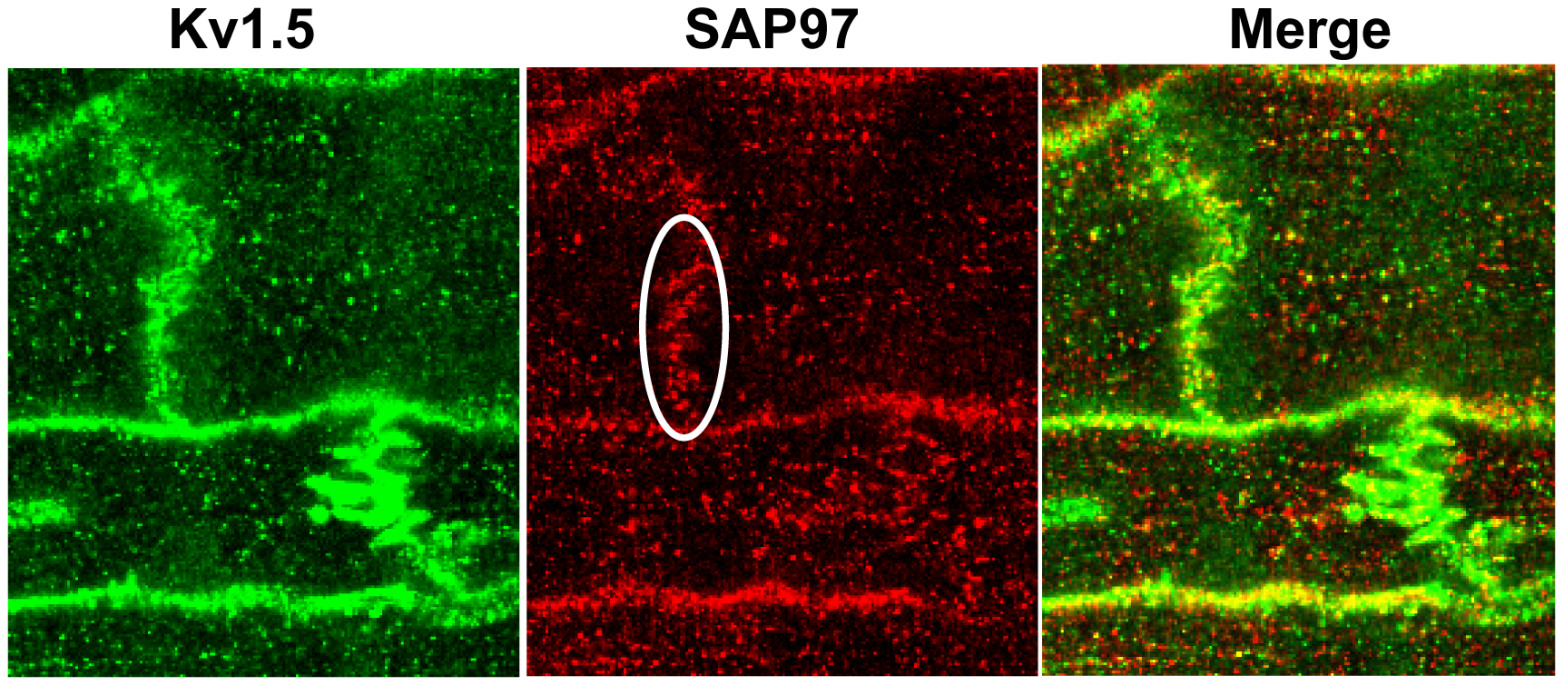
Co-immunolocalization of Kv1.5 (green) and SAP-97(red) in an aggregate of NZPCs.

**Figure 3:**
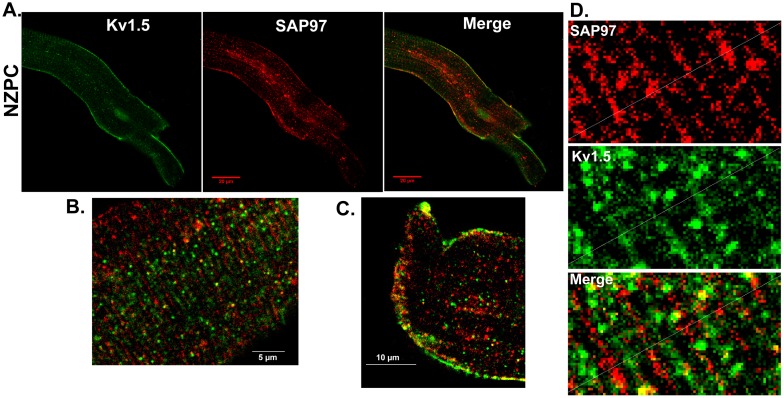
Co-immunolocalization of Kv1.5 and SAP-97 in a single NZPC (A). **B**, Costaining of the subcellular surface for Kv1.5 (green) and SAP-97 (red) in an NZPC. **C**, Localization of Kv1.5 (green) and SAP97 (red) in the cell ID area of an NZPC.**D**. Analyses of the merged image of Figure 3B illustrating the lack of true colocalization between SAP-97 (red) and Kv1.5 (green).Note the near absence of yellow signals. Scales were inset.

Single and aggregated PCs were stained for Kv1.5. **Figure 1** shows the location of the channel protein under these conditions. The NZPC channels show a high density at the intercalated disk (ID) (Panel A) with secondary, but minor, sarcolemmal staining. This result is similar to that for other non-t-tubule cardiac cells (e.g., atrial cells [9]). In the IZPCs (Panel B), the ID and sarcolemmal staining for Kv1.5 remains the same as for the NZPCs, but the cytosolic staining increases, perhaps reflecting the stress of the infarct process. Others have shown that stress alters Kv1.5 channels [10]. Under higher resolution microscopy (**Figure 2**), Purkinje cells show intense sarcolemmal and ID staining with some cytosolic puncta (green, left panel). Importantly, we find that SAP-97, a PDZ domain-containing and Nav1.5 channel-associated protein, is localized (red, mid panel) with Kv1.5 at *both* the ID and sarcolemmal locations (Merge panel). This colocalization may allow for increased Kv1.5 currents via an N-terminus mechanism [11]. Thus, unlike the results of Petitprez et al [12] and Abi-Char et al [13], in non-tubular NZPCs, Kv1.5 resides at IDs and sarcolemma where it cohabits with SAP-97 (**Figure 3**). Furthermore, while the signals overlap at IDs in an NZPC (**Figure 3C**), this overlap does not occur just below the cell surface in NZPCs (**Figure 3B**). In fact, at this magnification, both the Kv1.5 protein(green) and SAP-97 protein (red) of this NZPC show a striated alignment **without significant** colocalization (**Figure 3D**), which is in agreement with the results of other studies [14].

**Figure 4:**
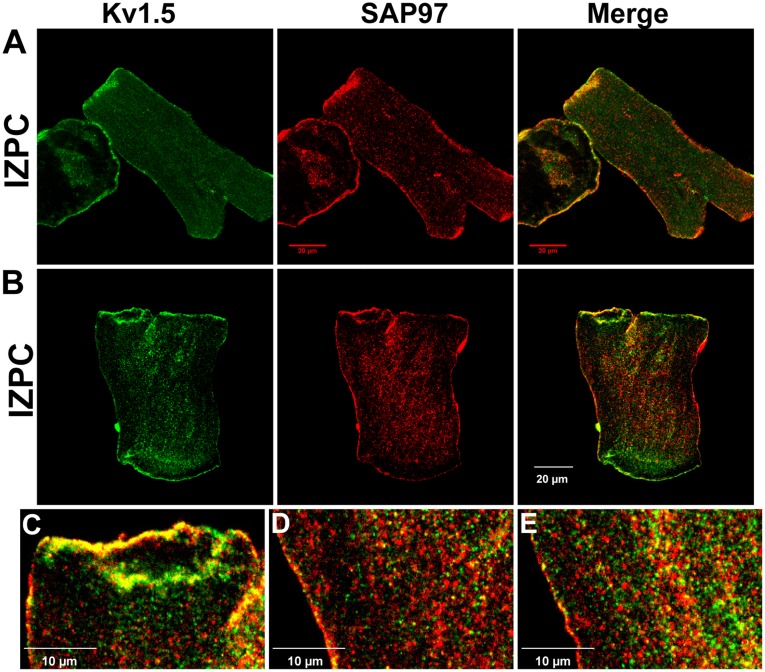
Immunolocalization of Kv1.5 and SAP-97 in IZPCs (A and B). **C**, Enlarged image showing the colocalization of Kv1.5 and SAP-97 in IDs. **D**, Enlarged image showing the localization of Kv1.5 (green) and SAP-97 (red) in the center of an IZPC. **E**, Enlarged image showing the localization of Kv1.5 (green) and SAP-97 (red) at the subcellular surface of an IZPC. Compare to Figure 3B. Scales were inset.

**Figure 5:**
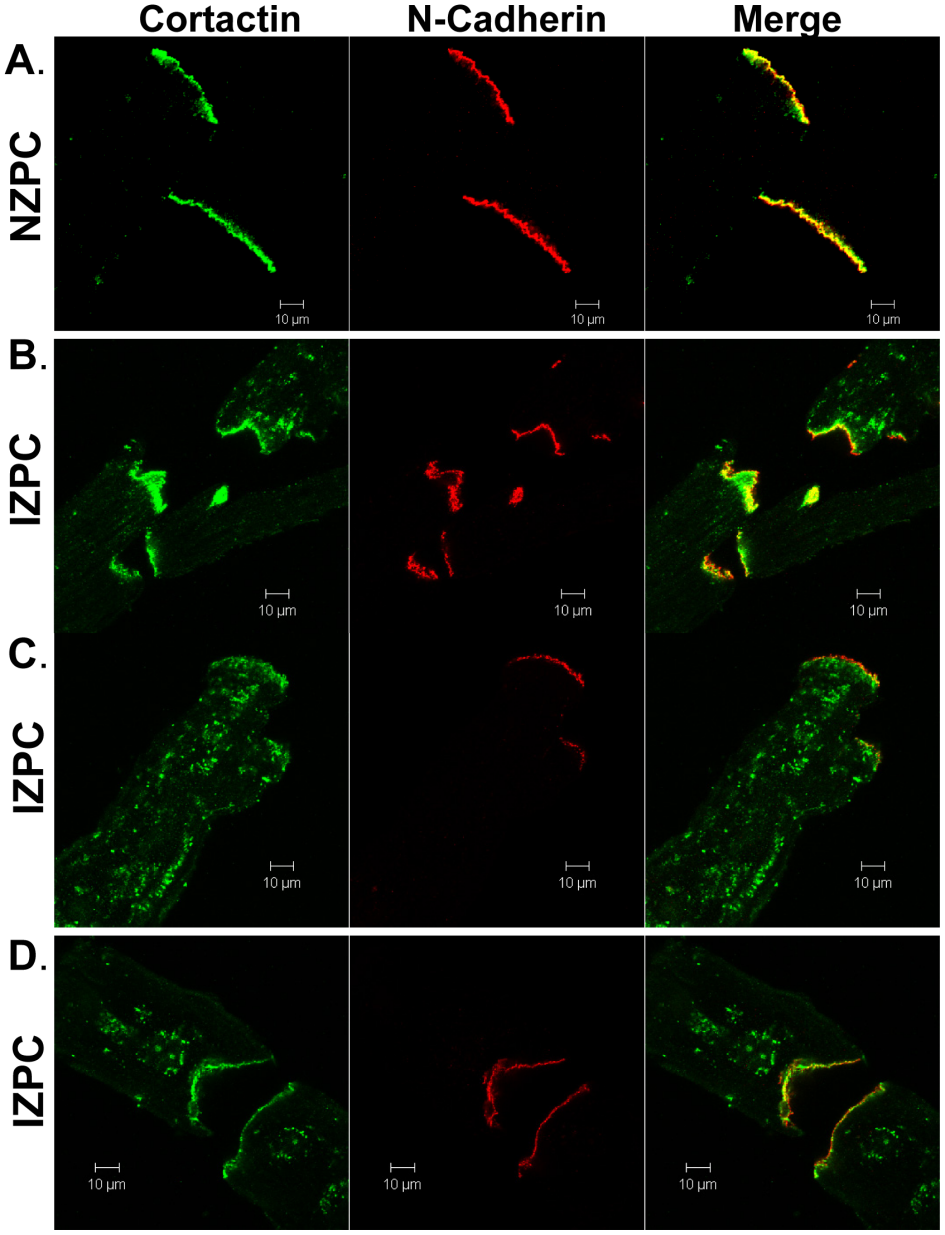
Immunolocalization of cortactin and N-cadherin in an NZPC (A) and several IZPCs (B, C and D). Horizontal scale = 10 µm.

**Figure 6:**
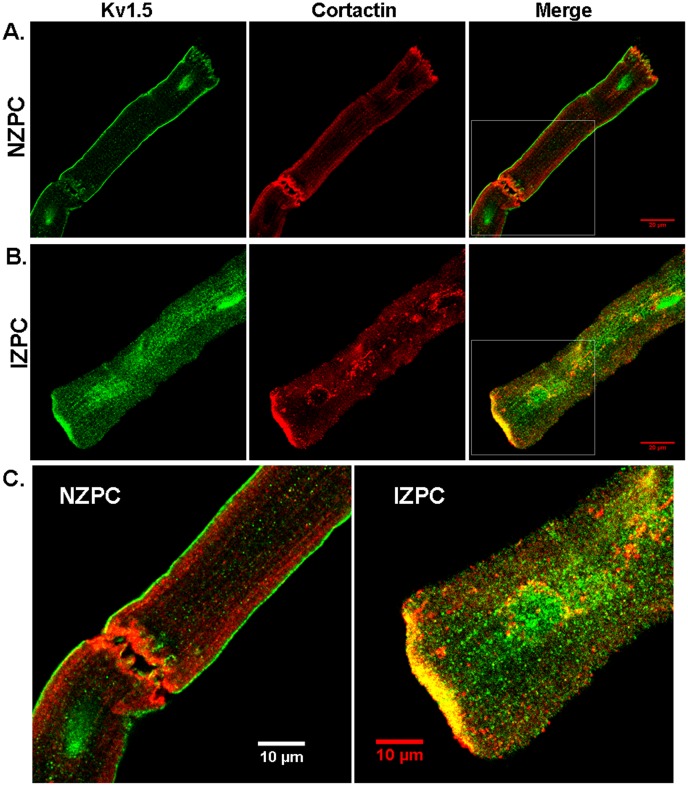
Immunolocalization of Kv1.5 and cortactin in an NZPC (A) and IZPC (B). Enlarged images from the area of the white rectangles are shown in Panel **C** for NZPC (left) and IZPC (right). Scales were inset.

In IZPCs, we observed variable staining (see 3 IZPCs in **Figure 4A–B**). In IZPCs, SAP-97 (red) was present at the sarcolemma; however, the Kv1.5 immunosignal (green) is augmented at the IDs in some cells. Changes in SAP-97occur at a time when N-cadherins have not changed in IZPCs vs NZPCs (**Figure 5** **, middle panels** (3 IZPCs and 1 NZPC; see also [15]). Little change is observed in the N-cadherin (middle columns) at the IDs of the IZPCs; however, cortactin, an actin-binding protein that is thought to associate with Kv1.5 channels [16], is markedly altered in IZPCs (**Figure 5B, C, D left panel** **s**). In NZPCs, cortactin is located with N-cadherin at IDs and just below the SL (**Figures 5A** **,** **6A**) but not in the perinuclear areas. In IZPCs, cortactin remains at the IDs but has migrated away from the ID in some IZPCs (**Figure 6B**) to form visible aggregates throughout the IZPC (**Figure 5C** **)**. In some of the same IZPCs, cytosolic cortactin forms ring structures (**Figure 5D** **,** **6B**). Because cortactin associates with Kv1.5 proteins [16], we determined that the ringed cortactin in the cytosol of IZPCs colocalizes with Kv1.5 around a nuclear area (**Figure 6C**). In NZPCs, there is no association between cortactin and nuclear Kv1.5 (**Figure 6C**).

### Electrophysiologic impact of Kv1.5 and associated proteins on I_Kur_ in IZPCs vs NZPCs

**Figure 7:**
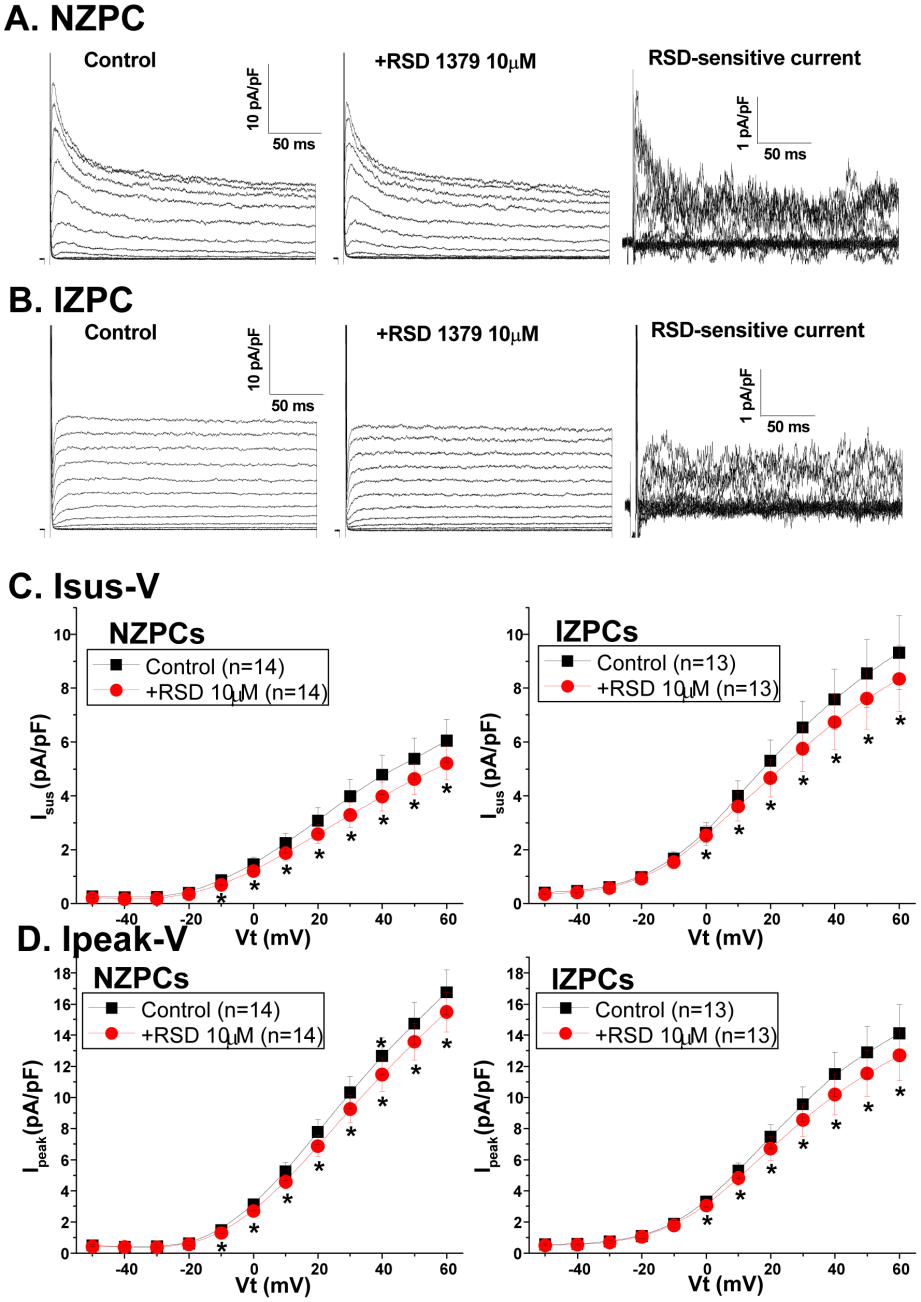
Current traces for an NZPC (A) and an IZPC (B). The cell was held at Vh = −60 mV and clamped in 10-mV increments to various test potentials. Traces were first obtained in the control solution (left), followed by a solution containing 10 µM RSD1379 for 7–9 min (middle). The currents sensitive to 10 µM RSD1379 were then obtained and are shown in the right panel. Plots of the current density-voltage relations in the absence and presence of 10 µM RSD 1379 for both Isus (**C**) and Ipeak (**D**) from NZPCs and IZPCs. RSD1379 significantly inhibited Isus and Ipeak in both NZPCs and IZPCs.

**Figure 8:**
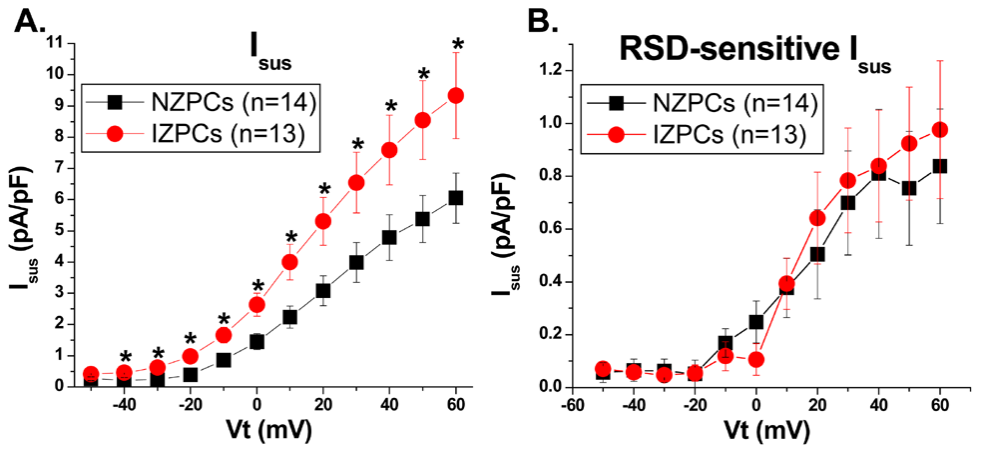
Basal Isus I-V curves (A) and RSD-sensitive Isus I-V curves (B) in NZPCs and IZPCs. The density of the basal Isus differed significantly between the NZPCs and IZPCs for all Vt>−40 mV. There was no significant difference in the RSD-sensitive Isus density between NZPCs and IZPCs.

In mammalian atrial cells, Kv channels must cluster to have robust Kv1.5-dependent currents, and this protein underlies the ultrarapid K current, I_Kur_. Recently, molecules have been developed that selectively block this channel. In this experiment, we used 10 µM RSD1379 to measure the Kv1.5 current; this compound that blocks Kv1.5 by 50% at 4.4 µM concentrations and has no or minor effects on other channels (Kv3.1, Kv2.1 and Kv4.2) at 10 µM [18]. RSD1379 blocked both the peak and sustained outward currents in both NZPCs and IZPCS (**Figure 7**). However, the RSD-sensitive currents did not differ in the two cell types (**Figure 8**). The RSD1379-sensitive currents at +40 mV did not differ between NZPCs (0.81±0.24 pA/pF, n = 14) and IZPCs (0.83±0.21 pA/pF, n = 13, NS) (**Figure 8B**) despite IZPC changes in drug-sensitive outward currents similar to those described in [3]. Therefore, despite the remodeling of significant proteins, there is no effect on the whole-cell functional I_Kur_ in Purkinje cells from the infarcted heart.

## Discussion

This report is the first to describe the existence of the KCNA5 protein in normal and diseased Purkinje cells. When secured in the sarcolemma (SL), the KCNA5 protein produces an outward K current; for both murine and human atrial cells, this current has been described as ultrarapid (I_Kur_ current). Indeed, the PC currents blocked by the selective blocker RSD1379 rapidly activate and inactivate with an IV curve similar to that in atrial cells. In fact, the NZPC Kv1.5 channel protein is present at both the SL and intercalated disk regions, a finding that is similar to reports in other cardiac cell types [9], [17], [19]. However, in IZPCs, the staining for both SL and ID Kv1.5 protein is intense, and the amount of cytosolic Kv1.5 protein is greatly increased. In NZPCs, some cells contained weak signals for Kv1.5 in nuclear regions (Figure 3). At a higher resolution, the Kv1.5 protein appears to show a striated pattern at the cell surface of an NZPC (Figure 3B). This pattern is not found in IZPCs (Figure 4C). Thus, subcellular structural remodeling of the *KCNA5* protein has occurred in arrhythmogenic IZPCs within 48 hr of coronary artery occlusion.

One anchoring protein that has been linked to Kv1.5 and its ability to form membrane clusters and thus enhance outward currents is SAP-97, a synapse-associated protein [12], [13], [19] [20], [21]. Published data suggest that colocalization of Kv1.5 and SAP-97 occurs predominately at the ID, similar to the colocalization with other K channels (e.g., Kv4.3 [22]). We show here that in NZPCs, Kv1.5 and SAP-97 *appear* to colocalize (Figure 2) at specific but not all sections of IDs but *also* along the SL. In single NZPCs, Figures 3B and D show that this is not true colocalization because the striated pattern of the red signals (SAP-97) does not overlap with the striated pattern of the Kv1.5 protein (green). The IDs in the NZPCs again show some overlap of these signals (Figure 3C). In IZPCs, the amount of SAP-97 is increased at IDs, but SAP-97 also has a notable cytosolic localization. However, under our conditions, there is little overlap of the two signals in the cytosol. Thus, an auxiliary protein that has been shown to regulate the functional expression and stabilization of Kv1.5 channels is clearly remodeled in IZPCs.

Another protein thought to control the surface expression of another K channel (Kv10.1) [23] is cortactin. Cortactin links ion channels with the cytoskeleton and serves as a platform that helps to integrate regulators of the actin assembly at the zona adherens of IDs [24]. One report has shown that cortactin co-immunoprecipitates and colocalizes with Kv1.5 in murine cardiac cells [16]. Furthermore, these authors suggest that cortactin is required for the N-cadherin regulation of Kv1.5 channel function because the knockdown of cortactin removed the N-cadherin-induced increase in Kv1.5 currents. We observed that cortactin and N-cadherin overlap almost entirely in NZPCs (Figure 5A), but the same cells show little cortactin/Kv1.5 overlap at IDs (Figure 6A). Cortactin becomes dramatically remodeled in IZPCs (Figures 5,6). In fact, while the N-cadherins remain unaltered (see also [15]), cortactin begins to dissociate from N-cadherin in IZPCs (Figure 5B). In IZPCs, dissociated cortactin forms aggregates within the cell (Figure 5C). Some, but not all, aggregated cortactin colocalizes with the Kv1.5 protein (yellow in Figure 6B, C). At the same time, the cortactin/Kv1.5 colocalization increases at the ID (Figure 6C). Finally, cortactin forms ring structures around IZPC nuclei filled with the Kv1.5 signal (Figure 6B,C), suggesting an ongoing active process of membrane trafficking of the channel protein.

### Functional Effect of Subcellular Structural Remodeling of KCNA5, COR and SAP-97

Despite the remodeling of several Kv1.5-related proteins, the currents sensitive to the I_Kur_ blocker (RSD1379) do not differ between NZPCs and IZPCs. Despite the obvious phenotypic differences between the K currents of IZPCs and NZPCs without drug (Figure 7; see also [1] [2]), the sustained RSD-blocked current component did not differ between the two cell types. Thus, normal Purkinje cells have an I_Kur_ current, and this current is unchanged in IZPCs, suggesting that the above mentioned protein remodeling is part of a new equilibrium. Therefore it is unlikely that altered I_Kur_ function plays a role in the abnormal APs of IZPCs [1].

### Remodeled Kv1.5 in Other Diseases

Both the subcellular structural and electrical remodeling of the Kv1.5 channels in atrial cells have been studied for many years. In these studies, atrial cells from patients with atrial fibrillation have altered Kv1.5 (decreased Kv surface expression and reduced I_Kur_ currents), and this alteration occurs along with cytoskeletal changes [25]. Furthermore, mutation or disruption of Kv1.5 trafficking via the cytoskeleton has been related to the onset of AF in mice [26]. Some of the proteins involved in the trafficking of this channel have been studied recently [27], [28]. In the work presented here, the involved proteins have clearly been remodeled, but the I_Kur_ like currents in Purkinje cells 48 hr post MI are not altered, suggesting that at least at this time point post MI, the mechanisms promoting endocytosis and those promoting the membrane insertion of the channel are in a new equilibrium. Alternatively, the associated proteins studied do not modulate total Purkinje I_Kur_ in a fashion described previously for myocytes.

### Limitations

This study focused on one K^+^ current which by this study we now know is in canine Purkinje cells; that is, the protein KCNA5 and I_Kur_ current. While super resolution confocal microscopy or co-IPs can be used to evaluate nanometer protein-protein associations and interactions, we did define subcellular colocalization domains in Purkinje cells from both normal and diseased hearts using our rigorous confocal methods. Despite the fact that co-IPs were not possible due to the limited amount of Purkinje study material, we did define a relationship between SAP-97 and cortactin proteins in the subcellular distribution of KCNA5 in IZPCs during post MI remodeling. However, we did not find a loss or gain of function of I_Kur_ in IZPCs suggesting these auxiliary proteins may be playing a role in other aspects of post MI remodeling.
